# Strategies to Potentiate Paracrine Therapeutic Efficacy of Mesenchymal Stem Cells in Inflammatory Diseases

**DOI:** 10.3390/ijms22073397

**Published:** 2021-03-25

**Authors:** Yoojin Seo, Min-Jung Kang, Hyung-Sik Kim

**Affiliations:** 1 Dental and Life Science Institute, Pusan National University, Yangsan 50612, Korea; amaicat24@naver.com (Y.S.); kkang085@naver.com (M.-J.K.); 2 School of Dentistry, Pusan National University, Yangsan 50612, Korea

**Keywords:** mesenchymal stem cells, immunomodulation, bioengineering, cell therapeutics

## Abstract

Mesenchymal stem cells (MSCs) have been developed as cell therapeutics for various immune disorders using their immunoregulatory properties mainly exerted by their paracrine functions. However, variation among cells from different donors, as well as rapid clearance after transplantation have impaired the uniform efficacy of MSCs and limited their application. Recently, several strategies to overcome this limitation have been suggested and proven in pre-clinical settings. Therefore, in this review article, we will update the knowledge on bioengineering strategies to improve the immunomodulatory functions of MSCs, including genetic modification and physical engineering.

## 1. Introduction

Mesenchymal stem cells (MSCs) are the multipotent adult stromal cells that can self-renew and differentiate into various cell types of the mesodermal lineage. Moreover, MSCs have been revealed to possess unique immunomodulatory properties through a broad spectrum of mechanisms including cell-to-cell contact and mediation of soluble factors [1,2], rendering them an attractive candidate for cellular therapy for a wide range of immune-related diseases. Indeed, a variety of innate immune cells including monocytes/macrophages, dendritic cells (DC), natural killer (NK) cells and adaptive immune cells such as T cells and B cells are polarized to the inactive or inflammation-resolving state by MSCs [3]. In particular, growing attention has been paid to the paracrine capacity of MSCs in this context since several MSC-derived paracrine factors are associated with their immunomodulatory effects [4]. For instance, indoleamine 2,3-dioxygenase (IDO) and prostaglandin E2 (PGE2) derived from MSCs participate in the suppression of pro-inflammatory macrophage polarization, T cell proliferation and NK cell cytotoxicity [5,6], while MSC-derived transforming growth factor-β (TGF-β) leads to the systemic immune tolerance via inducing the regulatory T cells [7].

At present, hundreds of clinical trials have been conducted to treat immune-mediated disease with MSCs; however, the clinical application of MSCs often fails to recapitulate therapeutic potential for immunomodulation despite the promising results from in vitro and animal studies, partially due to their heterogeneity according to originated sources and diversity of delivery methods (e.g., cell dose, injection route and treatment frequency) [8]. Dynamic interaction between transfused MSCs and unfavorable host microenvironments such as nutrient deficiency, hypoxia and extensive inflammatory stimuli also changes the characteristics of MSCs, reducing the benefit of cell therapy [9]. Therefore, strict quality management of MSCs and standardization of their potency in vivo must precede the practical application to achieve reproducible and successful clinical outcomes as demonstrated in the preliminary settings [10,11]. In addition, it is necessary to explore novel strategies to strengthen the therapeutic capacity of MSCs. This review summarizes the state-of-the-art engineering technologies for the clinical translation of MSCs, with a focus on enhancing their paracrine activity.

## 2. Immunomodulatory Property of MSCs

### 2.1. Interaction between MSCs and Innate Immune Cells

#### 2.1.1. Macrophage

Macrophage are the crucial cell component in the innate immune system with significant plasticity. The activation state of macrophages can be divided into two directions: classically polarization towards M1 (pro-inflammatory subtype) or alternatively activated M2 type (anti-inflammatory subtype) [12]. In general, MSCs tend to inhibit M1 type while inducing M2 type, contributing to the resolution of inflammation and further tissue regeneration [13]. In this context, MSC-derived paracrine factors such as indoleamine 2,3-dioxygenase (IDO) and prostaglandin E2 (PGE2) play a significant role [14]. In addition, transforming growth factor-β (TGF-β) pathway is involved in the M2 macrophage differentiation process induced by MSCs [15].

#### 2.1.2. Myeloid Dendritic Cells (DCs)

DCs play as antigen-presenting cells (APCs), linking the innate- and adaptive immune system cascade [16]. MSCs can compromise their APC function via the suppression of differentiation, maturation and migration of DCs; MSCs inhibit the differentiation of monocytes to DCs by suppressing their expression of Major Histocompatibility Complex II (MHC II), CD1-α, CD80 and CD86 and IL-12 [17]. Similar to macrophage polarization, MSCs can induce DCs into an anti-inflammatory phenotype through downregulating the pro-inflammatory factors (i.e., TNF-α and IL-12) and upregulating the anti-inflammatory factors (i.e., IL-10) in DCs [18]. In addition, MSCs prevent LPS-mediated maturation of DCs and induces regulatory DC population in a hepatic growth factor (HGF)-dependent manner [19].

#### 2.1.3. Natural Killer (NK) Cells

Natural killer (NK) cells are the key effector cells possessing both cytotoxic lymphocyte function and anti-tumor/anti-viral capacity in the innate immune system [20,21]. Notably, MSCs exert potent inhibitory functions on NK cell proliferation, differentiation and migration and these suppressive impact of MSCs on NK cells are mediated by MSC-derived general immunomodulating factors including IDO, PGE2 and human leukocyte antigen-G5 (HLA-G5) [22,23]. Interestingly, NK cell-derived IL-12- or IL-18 promotes IFN-γ secretion, which would further enhance C-C Motif Chemokine Ligand 2 (CCL2) expression and immunomodulatory capacity of MSCs [24].

#### 2.1.4. Neutrophils

Neutrophils are abundantly found in the bloodstream and are regarded as the key players during acute inflammation [25], defending the invading microorganisms, while they also function as APCs to trigger the adaptive immune response [26]. MSCs provide some protective effects on neutrophils against apoptosis and promote their recruitment to the bone marrow through the recreation of IL-6, IL-8 and macrophage migration inhibitory factor (MIF) [27]. On the contrary, some reports have shown that MSCs play the opposite role since they would impede neutrophils’ recruitment and functionality in terms of extracellular trap formation and protease secretion by secreting superoxide dismutase-3 (SOD-3) [28]. Thus, neutrophils modulation by MSCs might depend on the immunophenotype of MSCs.

### 2.2. Interaction between MSCs and Adaptive Immune Cells

#### 2.2.1. T Cells

In general, MSCs suppress T-cell proliferation (both CD4+ and CD8+ T cell subsets) and activation regardless of their originated species and tissue types. As mentioned in Section 2.1.2., MSCs inhibit DC maturation and, in turn, reduce the T cell activation. MSCs expressing HLA-G1 and TGF-β inhibit T-cell proliferation by reducing cell-cycle associated components such as phospho-retinoblastoma (pRb), cyclin D and cyclin A, resulting in growth arrest in the G1 phase [29]. PD-L1 secreted by MSCs also acts on T cell apoptosis and influences an irreversible T cell hypo- responsiveness [30]. In terms of helper T cell subtype, MSCs can interfere with the differentiation of pro-inflammatory Th1 to anti-inflammatory Th2 condition [31]. MSCs inhibit Th1 type pro-inflammatory factor expression (I.e. IFN-γ, TNF-α and IL-1β) and induce an increase in IL-10 levels and thus, enhancing Th2 type factor expression. MSCs also inhibit the differentiation and function of Th17 cells by de-activating the signal transducer and activator of transcription 3 (STAT3) pathway through PD-1, IL-10, CCL2 or suppressor of cytokine signaling 3 (SOCS3) expression [32]. MSCs are known to directly induce the differentiation of regulatory T cells (Treg), T cells mainly involved in T cell suppression and immunomodulation for maintenance of homeostasis [33], through the TLR-Notch pathway and the secretion of IL-10, TGF-β1, IDO and inducible nitric oxide synthase (iNOS) [34]. In addition, MSCs suppress the secretion of pro-inflammatory cytokines including interferon-γ (IFN-γ), IL-22 and IL-17, but promote IL-10 production by Th1 and Th17 cells, inducing the generation of Treg [35].

#### 2.2.2. B Cells

MSCs affect differentiation, proliferation, reduce cell cycle arrest, impaired plasma cell generation and compromised the immunoglobulin-secreting ability of B cells [36]. MSCs inhibit STAT3 activation and induces PAX5 expression via CCL2 secretion to suppress immunoglobulin synthesis in B cells [37]. MSCs secrete IL-1 receptor antagonist (IL-1Ra) and PD-L1 to inhibit B-cell differentiation [38]. Finally, MSCs drive the induction of regulatory B cells (Bregs) or naïve B cells with memory function and IL-10 derived from the Breg further enhances the immunomodulation of MSCs via converting effector T cells into Tregs [39,40].

### 2.3. MSC-Derived Immune Modulators

MSCs display immunomodulatory phenotype partially via the secretion of immune-associated bioactive factors depending on the context of the microenvironment. These soluble factors include a diverse multitude of cytokines, growth factors, chemokines and hormones, which combine to modulate the immune system [41].

PGE2 is regarded as one of the most potent, key immunosuppressive factors of MSCs. It is generated from the arachidonic acid in the membrane phospholipids by cyclooxygenase-1 and 2 (COX-1 and COX-2) and prostaglandin synthase [42]. MSC-derived PGE2 modulates the direction of macrophage polarization from the pro-inflammatory phenotype M1 into the anti-inflammation phenotype M2 and exerting the inhibitory effects of MSCs on DCs by inducing up-regulation of IL-10 secretion from DCs [43]. In addition, PGE2 inhibits T cell proliferation, Th17 cell differentiation and NK cell cytotoxicity [44].

MSC-derived IDO plays immunosuppressive roles against various immune cells, including Macrophages, DCs, T cells and NK cells [6]. IDO catalyzes the conversion of tryptophan to kynurenine, which inhibits the proliferation of immune cells. IDO leads to T cell suppression by skewing the pro-inflammatory Th1 state to the anti-inflammatory Th2 condition [6]. IDO secretion by MSCs inhibits NK cell activation as well as the maturation of DC and M1 macrophages [45].

Finally, MSCs express iNOS, which metabolizes L-arginine into citrulline and produces NO, which suppresses the secretion of pro-inflammatory cytokines and T cell proliferation [46]. Upon exposure to pro-inflammatory cytokines in vitro, MSCs produce high amounts of NO to suppress the proliferation and modulation of T cells and other immune cells [47,48].

## 3. Clinical Application of MSCs for Immune-Associated Disorders

Given that MSCs exhibit an impressive immunomodulatory role in a context-dependent manner in pre-clinical settings, the practical efficacy of MSC application has been evaluated over the past decade. Statistically, bone-marrow (BM) is a dominant source for MSCs, while umbilical cord (UC) and adipose-tissue (AD)-derived MSCs are also frequently used in clinical trials [49]. The injection route is largely divided into intravenous systemic delivery and local delivery (i.e., intrathecal, intramuscular and intra-articular injection) [11,49]. The local injection of MSCs enables direct targeting of the problematic lesion but it is usually invasive and impedes the viability as well as engraftment of MSCs due to the harsh microenvironment [11]. On the contrary, systemic administration of MSCs can be a feasible option in various clinical circumstances and provides flexibility in terms of injection volume, dose and frequency. The major drawback of the intravenous route is the insufficient migration of MSCs to the target lesion. Indeed, most of the intravenously introduced cells are trapped in the lung and cleared by resident macrophages, which significantly dampens the therapeutic efficacy of MSC application [50,51]. In addition, undesirable immune responses so-called “instant blood-mediated inflammatory reaction” triggered by systemic MSC transplantation can elicit safety concerns [51]. Therefore, intensive monitoring of the injected cell fate as well as host response should be followed to overcome the current limitation and improve the therapeutic as observed in the preclinical investigation.

To mitigate and/or control the disabilities in the immune system with MSC application, several clinical trials targeting various intractable autoimmune disorders and inflammatory diseases such as graft-versus-host disease (GvHD), multiple sclerosis (MS), inflammatory bowel diseases (IBD) and systemic lupus erythematosus (SLE) have been conducted in MSC therapeutic felid (Table 1).

### 3.1. Graft-Versus-Host Disease (GvHD)

GvHD is a major cause of death after hematopoietic stem cell transplantation (HSCT) and is the result of donor-derived hematopoietic stem cell mounting an alloreactive response against host tissues and organs. GvHD is characterized by the immune response of helper T cells, showing the typical characteristics of autoimmune diseases [52]. Owing to the innate supporting and immunomodulatory role of MSCs for HSCs in the bone marrow, transplantation of MSCs has been applied to manage this complication and a total of 46 studies have been enrolled for the clinical trials (to February 2021, clinicaltrials.gov. 6 March 2021).

### 3.2. Multiple Sclerosis (MS)

MS is an autoimmune disorder with chronic, progressive inflammation in the central nervous system. The etiology is unknown, but autoimmune responses mainly of CD4+T cells that migrate from the periphery attack myelin-based protein, leading to demyelination and, in turn, neurodegeneration [53]. Since MSC application in the EAE model, the representative MS-recapitulating animal model, has been proven effective with promising outcomes, about 50 clinical trials have been conducted to estimate the therapeutic role of MSCs for treating MS (to February 2021, clinicaltrials.gov. 6 March 2021).

### 3.3. Inflammatory Bowel Diseases (IBD)

IBD is a chronic inflammatory disorder of the gastrointestinal tract associated with multifactorial conditions, such as ulcerative colitis (UC) and Chrohn’s disease (CD) [54]. The development and progression of IBD is influenced by numerous factors, such as the dysfunction of mucosal T cells, impairment in the mucosal/epithelial barrier, intestinal infections and dysbiosis [55]. The paracrine functions of MSCs can remedy these complications in various ways. A total of 34 clinical trials to treat IBD with MSCs have been conducted so far (to February 2021, clinicaltrials.gov. 6 March 2021). MSCs are provided IL-12 and TGF-β to control the function of NK cells and restrained the proliferation of B lymphocytes via promoting the expression of CD40 in colitis [56,57]. Nod-like receptor signaling pathway would be activated by MSCs to boost the PGE2 expression and reduce the multiplication of monocyte [58]. MSCs also secrete TGF-β to transform the phenotype of macrophages from M1, identified as pro-inflammatory properties, to M2. In the meantime, MSCs were able to secret TGF-β and IL-10 to inhibit the T cell activation and promote regulatory T cells [59]. Therefore, the therapeutic actions of MSC-paracrine factors are largely dependent on their immunomodulatory capacity in IBD.

### 3.4. Systemic Lupus Erythematosus (SLE)

Systemic lupus erythematosus (SLE) is a chronic autoimmune disease characterized by activation of B and T lymphocytes [60]. SLE is accompanied by the formation of immune complexes, tissue inflammation in multiple organs and high levels of serum pro-inflammatory cytokines. In addition, Tregs and T helper 17 cells play important roles in the pathogenesis of SLE [61]. So far, about 15 cases of clinical trials for SLE have been performed with MSC application (to February 2021, clinicaltrials.gov. 6 March 2021). Recent clinical studies have revealed that UC MSCs up-regulate Foxp3 + Treg cell and down-regulate Th17 cells through the regulation of TGF- β and PGE2 in SLE [44].

**Table 1:** Examples of clinical trials for the treatment of immune-mediated diseases using MSCs.

Disease	Origin of MSCs	Clinical Trial Number	Phase	MSC-Derived Soluble Factors	Alteration in the Immune System	References
GvHD	Allogeneic MSCs	NCT01522716	II	CXCL9 ↑ CXCL10 ↑	Naïve CD4 Tcell ↑ Naïve B cell ↑	[62]
IBD	Autologous MSCs	NCT01659762	I	IDO ↑	PBMC proliferation ↓	[63]
	Autologous BM-MSCs	NCT01659762	I	IL-10 ↑	Treg induction ↑ T cell apoptosis ↑	[64]
MS	Autologous BM-MSCs	NCT01228266	II	N.A	Th1/Th17 ratio ↓ Breg induction ↑	[65]
SLE	Allogeneic UC-MSCs	NCT01741857	I	TGF-β ↑ PGE2 ↑	Th17 cell proliferation ↓ Treg induction ↑	[44]

MSCs; mesenchymal stem cells, GvHD; Graft-versus-host disease, CXCL; Chemokine (C-X-C motif) ligand, IBD; Inflammatory bowel diseases, IDO; Indoleamine 2,3-dioxygenase, BM; bone marrow, PBMC; Peripheral blood mononuclear cell, IL; Interleukin, Treg; regulatory T cell, MS; Multiple sclerosis, N.A; not available, Breg; regulatory B cell, SLE; Systemic lupus erythematosus, UC; umbilical cord, TGF; Transforming growth factor, PGE2; Prostaglandin E2.

## 4. Bioengineering of MSCs for the Functional Improvement

Based on the improved understanding of mode-of-action underlying the MSC-mediated immune regulation as well as practical limitations of naïve cells, various bioengineering strategies aiming to maximize the therapeutic potency have been proposed [66,67]. These approaches can be briefly divided into (1) enforcement of innate paracrine function via priming or genetic engineering of MSCs and (2) biomaterial-based physical/structural modification of MSCs.

### 4.1. Enforcement of Innate Paracrine Function

#### 4.1.1. MSC Priming

Since the immunomodulatory function of MSCs is conferred by reciprocal communication with immune cells, pre-conditioning of MSCs with immune response mediators in vitro, so-called “priming” or “licensing” strategy, has been applied to enhance their innate immunomodulatory capacity [68,69]. IFN-γ, TNF-α and several interleukin families are the most frequently used bioactive agents and exposure to these pro-inflammatory cytokines prior to in vivo application can educate MSCs to acquire immunosuppressive phenotype via reinforcing their paracrine capacity mainly for IDO, PGE2, IL-10, TGF-β and NO [70]. Global transcriptome- and proteome analysis of MSCs has further revealed that activity of immune-associated key signaling such as NF-kB, JAK-STAT1/3, COX-2 and mTOR pathway can be dramatically altered upon priming towards anti-inflammatory signature [71,72,73,74]. As a result, the superior therapeutic performance of primed MSCs compared to naïve cells has been reported in various immune-related disorders including atopic dermatitis [75], experimental colitis [76], experimental autoimmune encephalomyelitis (EAE) [77], hepatic infection [78,79] and GvHD [73,80].

In addition, recapitulating the infectious condition in MSCs via stimulating the innate immune systems contributes to boosting their immune regulatory functions [81]. The pattern recognition receptors (PRRs) participate in the early response of innate immune cells by detecting the specific endogenous and exogenous dangerous signals. Of interest, MSCs are constantly expressing the PRRs such as TLRs and Nod-like receptors (NLRs) and PRR activation in MSCs can drive anti-inflammatory downstream response both in vitro and in vivo [82]. For example, MSCs cultured with TLR3 agonist polyinosinic:polycytidylic acid (poly I:C) or TLR4 agonist lipopolysaccharide (LPS) could suppress T cell proliferation and Th1/17 polarization to a greater extend to control cells partially via the activation of Notch pathway, resulting in a better clinical outcome in EAE and colitis model [33,83,84]. LPS primed MSCs exerted an enhanced innate antibacterial activity than naïve cells and promoted the faster bacterial clearance in septic mice [85]. Similarly, pre-activation of the NOD2 pathway using muramyl dipeptide (MDP) promoted the anti-inflammatory signature of MSCs mainly through the activation of COX-2 signaling and PGE2 secretion, which could ameliorate the disease severity of the experimental colitis model [58]. The potential role of innate immune sensor “inflammasome complex” in the regulation of MSC immunophenotype has been also demonstrated recently [86]. After the stimulation of NLRP3, one of the best-described inflammasomes in present, the immunomodulatory function of MSCs was potentiated in terms of induction of Treg as well as suppression of pro-inflammatory macrophage and NLRP3-activated MSCs could provide superior protection against colitis mice.

Although MSC priming is the foremost and simplest way to augment the MSC-derived immunoregulatory potential, several practical challenges remain prior to its clinical application. First, the intrinsic immunomodulatory nature of MSCs and their response to licensing agents vary depending on the cell origin and the priming protocols such as stimulant combination, treatment concentration and exposure time [87,88]. Moreover, the boosting impact of pre-conditioning often fails to reach a substantial level in vivo, resulting in unexpected therapeutic differences. Several advanced approaches have been suggested to overcome these limitations of priming strategy. One way is to utilize biomaterials for the fabrication of microparticle (MP), a bioinstructive molecule-carrying platform, to deliver the priming agents to the MSCs consistently [89]. Using this technique, licensing molecules can be anchored to MSCs. For instance, MSCs mixed with IFN-γ loaded-heparin MP presented a sustained expression of IDO and T cell suppressive property compared to traditionally-primed cells [90]. Moreover, MP containing the immune-response controlling chemicals can be internalized into MSCs to change their immunophenotype directly. Ranganath et al. delivered MPs encapsulating TPCA-1, an inhibitor of nuclear factor kappa-B kinase subunit-β (IKK-β), to MSCs. As MPs were internalized in cells, the intracellular release of TPCA-1 led to a stable inhibition of NF-kB pathway, preventing the unexpected pro-inflammatory response of MSCs upon TNF-α treatment [91]. Based on the prior finding that activation of glucocorticoid pathway augments the immunomodulatory function of MSCs, Ankrum et al. conducted MSC modification with MP carrying a glucocorticoid steroid budesonide [92]. Budesonide MP was efficiently internalized into MSCs and enhanced immunomodulatory potential along with stable IDO activity in vitro. Thus, MP-based local delivery of the bioactive compound to MSCs would be an effective and safe strategy to control the therapeutic capacity that can replace the conventional priming strategy.

#### 4.1.2. Genetic Engineering of MSCs

Although MP-mediated priming can provide a more constant and durable boosting impact on the paracrine capacity of MSCs, it can only potentiate the innate function. Therefore, researchers have applied genetic engineering techniques for the direct induction of either insufficient endogenous factors or brand-new proteins within MSCs [93]. In general, RNA viruses such as lentivirus and retrovirus are the most commonly used viral vectors for gene transfer owing to their host-genome integration capacity. Virus-transduced cells display the permanent expression of the desired gene product, while potential safety issues such as mutagenesis and tumorigenesis should be carefully monitored prior to their clinical application [94]. On the contrary, DNA viruses including adenovirus and adeno-associated virus (AAV) provide transient but relatively safer gene delivery. The viral vector-based method provides high potent gene transfer with low cytotoxicity; however, viral vectors tend to elicit host immune response which might dampen their efficiency [95]. Genetic information can be also introduced to MSCs via non-viral method using physical (i.e., microinjection, electroporation) or chemical (i.e., calcium-phosphate nanoparticle) tools, although significant cytotoxic effects during the procedure as well as unstable gene expression with low efficiency limit its practical use [96].

The representative pre-clinical outcomes of genetically enhanced MSCs targeting immune-associated diseases are summarized in Table 2. At present, IL-10 is the most frequently chosen overexpression target in MSC genetic engineering since IL-10 acts as a powerful immunomodulatory factor for the resolution of excessive inflammation and tissue regeneration. Therefore, the potential therapeutic impact of IL-10 overexpressing MSCs has been demonstrated in various immune-mediated pathologic conditions including various neuro-inflammatory/degenerative diseases [97,98,99], acute liver allograft rejection [100] and lung injury induced by ischemia-reperfusion damage or LPS challenge [101,102].

In addition, IL-10, another important anti-inflammatory cytokine, IL-4, can be genetically delivered in MSCs to enhance the immunosuppressive role of naive cells targeting autoimmune disorders [103,104]. To increase homing capacity to the injury site, Silva et al. introduced granulocyte-Colony Stimulating Factor (GM-CSF) to MSCs (MSC^GM-CSF^) and evaluated their therapeutic roles in Chagas disease cardiomyopathy [105]. Compared to control MSCs, MSC^GM-CSF^ displayed a remarkable homing ability to the heart, sequentially leading to the recruitment of myeloid-derived suppressor cells (MDSCs) and Treg induction. Likewise, the therapeutic efficacy of genetically engineered IFN-β expressing MSCs (MSC^IFN-β^) was evaluated in murine EAE model owing to the beneficial role of recombinant IFN-ß in the management of MS [106]. The authors found that MSC^IFN-β^ led to a significant recovery of demyelination in the spinal cord accompanied by a reduction in clinical score of EAE mice, partially via the suppression of circulating CD25/69+ activated CD4+ T cells.

As represented by IL-1α and β, activation of IL-1 pathway is generally associated with clinical deterioration of inflammatory disease [118]; interestingly, however, some of the recently-discovered IL-1 family members such as IL-1Ra are known to suppress the classical pro-inflammatory IL-1 function and, thus, treatment of MSCs expressing these endogenous IL-1 antagonists can provide superior therapeutic benefits than naïve cells. Indeed, overexpression of IL-1Ra in MSCs improved the survival of the fulminant hepatic failure model by alleviating the liver damage accompanied by the attenuation of intrahepatic inflammation [107]. Inhibition of IL-1 signaling with MSC overexpressing IL-37 also augmented the anti-inflammatory capacity of MSCs both in vitro and in vivo, reducing the SLE-like symptoms in the mouse model [108]. Given that pro-inflammatory IL-33 binds to its receptor IL-33/IL-1 receptor–like–1 (ST2) to elicit the Th2 differentiation, González et al. genetically introduced soluble IL-1 receptor–like–1 (sST2) into MSCs to block the IL-33/ST2 interaction [109]. The authors found that sST2-expressing MSCs brought an improvement in the clinical severity of endotoxemia as well as histological pathology in a murine model of LPS-mediated lung injury, demonstrating the therapeutic benefits of targeting the IL-33 pathway for the management of the acute respiratory disease.

Meanwhile, microRNAs (miRNAs) are attractive overexpression targets for the functional improvement of MSCs. miRNAs are highly conserved single-stranded non-coding RNA molecules that induce gene silencing either by degradation or translational blocking of target messenger RNA. Importantly, cell-to-cell communication is largely mediated by the exchange of miRNAs-containing extracellular vesicles (EVs). In this context, MSCs and their EVs can be utilized as therapeutic miRNA delivering tools and miRNAs involved in immune regulation have been introduced to MSCs to upregulate the innate immunomodulatory function of MSCs. For instance, the introduction of miR-223, which can negatively regulate the pro-inflammatory responses such as activation of NLRP3 inflammasome pathway, can enhance the MSC-mediated protection against murine models of experimental autoimmune hepatitis [111]. Zilun et al. overexpressed T cell-regulating miR-181a in MSCs and found that exosomes derived from miR-181a overexpressing MSCs led to a prominent induction of Treg cells in injured cardiac tissue compared to control [112]. The therapeutic advantage of miR-181 overexpression in MSCs was also revealed in liver fibrosis model, in which MSCs could induce autophagy of hepatic satellite cells and down-regulate inflammatory response upon miR-181-5p overexpression [113]. In addition, clinical data-based miRNA selection is another commonly used strategy; indeed, given that the expression level of miR-30d-5p tend to be down-regulated in the serum of stroke patients, Jiang et al. generated miR-30d-5p overexpressing MSCs and demonstrated their protective impact on M1 microglia-mediated acute ischemic stroke injury [114]. Both in vitro and in vivo, MSC-derived miR-30d-5p skewed M2 polarization by preventing abnormal autophagy.

Overexpression of angiogenic- or pro-survival factors also augments the overall therapeutic efficacy of MSCs against inflammatory disease. For instance, angiopoietin 1, a major player for blood vessel formation and maturation, enhanced the benefits of MSCs in the acute lung injury (ALI) model by reducing vascular leakage [115]. The overexpression of superoxide dismutase 3 (SOD3) in MSCs resulted in the increment of cell viability both in vitro and in vivo, contributing to functional improvement of MSCs in various inflammatory skin disease models [116,117].

#### 4.1.3. CRISPR/Cas9-Based Functional Improvement of MSCs

In recent years, the groundbreaking technique called Clustered Regularly Interspaced Short Palindromic Repeats (CRISPR) system contributes to a profound development in the field of gene therapy [119,120]. The operating principle of CRISPR/Cas9-based gene editing is initiated with the induction of target-site specific double-strand breaks with Cas9 endonuclease to activate the DNA repair system, which in turn leads to gene correction or mutation. The advanced utilization of mutated Cas9 with Nickase activity enables researchers to perform more accurate and purpose-specific genomic engineering [121]. Moreover, deactivated Cas9 (dCas9) without catalytic activity applies to transcriptional regulation of the target genes, expanding the scope of the technique beyond genome editing [121]. Owing to its convenience and economic advantages compared to conventional methods, CRISPR has become the most popular genome engineering technique.

Growing attempts have been conducted to apply CRISPR/Cas9-mediated gene modification in the field of MSC therapeutics [122]. First, CRISPR-based gene silencing can modify the intrinsic nature of naïve cells. Shen et al. have shown that knockout of tumor suppressor phosphatase and tensin homolog (PTEN) in BM-MSCs via CRISPR/Cas9-mediated exon targeting increased the cell proliferative capacity accompanied with the decreased osteogenic- and adipogenic potentials compared to control cells [123]. In a recent work by Zha et al., authors have utilized CRISPR/Cas9 system targeting one of MHC class I molecules β2 microglobulin (B2M) to generate “less immunogenic” iPSC-derived MSC lines for the allogenic transplantation [124]. It is noted that B2M-KO MSCs could escape more efficiently from the immune response-mediated killing by peripheral blood-derived monocytes (PBMCs) than control cells, while the loss of B2M expression did not alter the innate immunosuppressive feature of MSCs. In addition, since CRISPR/Cas9 system only requires short nucleotides so-called guide RNAs (gRNAs) to recognize the target site, researchers can knock-in the desired sequences at a specific, intended “safe” location. For instance, Hu et al. have represented a concept of CRISPR-based cell immortalization strategy for mouse MSCs by introducing simian virus SV40 large T antigen into an intrinsic safe harboring site at Rosa26 locus [125]. In other reports, CRISPR/Cas9 construct was simultaneously delivered with the AAV vector then gene construct that encodes beneficial protein was knock-in into the AAV-specific safe locus such as AAVS1 [126,127]. This AAV-CRISPR/Cas9 genetic engineering platform can induce the stable overexpression of therapeutic factors in MSCs in a relatively simple and safe way. Finally, dCas9-based gene regulation at the transcriptional level can change the fate of MSCs. Indeed, CRISPR-mediated activation of the adipogenic system induced spontaneous adipogenesis in MSCs and switching the target gene combination could control the white/beige adipocyte ratio during the differentiation condition [128]. Sun et al. have reported that MSCs were transformed into sweat-gland like cells via ectopic stimulation of ectodysplasin promoter with dCas9 [129]. Moreover, dCas9-activation mediator system has been applied to generate IL-10-overexpressing MSCs, which could suppress the immune cell accumulation and pro-inflammatory response in the diabetes-associated myocardial infarction model [110]. In the future, CRISPR/Cas9 technique would contribute to enhancing the therapeutic potential of MSCs in immune-associated disorders not only by upregulating the beneficial immunomodulatory factors (via knock-in or transcriptional activation strategy) but also by lowering their immunogenicity (via knock-out strategy) to prevent host immune-rejection.

### 4.2. Structural/Physical Engineering of MSCs

#### 4.2.1. 3D Assembly of MSCs via Spheroid Formation

After the isolation and phenotype validation, MSCs are grown on a flat plastic surface as a monolayer sheet in general. This conventional 2D culture method is a well-established convenient system to obtain a large number of cells in a short time; however, it significantly affects the innate characters of MSCs and even diminishes their therapeutic potentials [130,131]. For instance, a standardized culture condition and fast expansion cycle lead to premature cellular senescence, lowering both cell yields and quality. Another major drawback of the 2D system is the lack of proper cell-to-cell and/or cell-to-microenvironment communications. In vivo, MSCs reside in the “stem cell niche” surrounded by other cell components and extracellular matrix (ECM) and depending on physio-pathological circumstances, as well as the tissue-of origin, dynamic nature within the niche such as concentration gradients of oxygen and nutrient, mechanical force changes and multiple paracrine signals from neighbor cells can regulate MSC behavior. On the contrary, 2D-cultured cells seem to lose their heterozygosity due to the limited cellular interaction and identical microenvironment supplemented with sufficient nutrients and constant oxygen level. Hence, established 2D MSC lines often fail to represent their in vivo response to various stimuli, hindering the accurate estimation of the therapeutic effect of MSC application in the practical field.

The disadvantages of the traditional method have led to the development of an advanced 3D cell culture system. For instance, floating cells in a small droplet (hanging drop culture) or centrifugation of cells in low-attachment wells (forced aggregation method) leads to the formation of the spheroid-like structure by gravitational force [132]. Since the aggregation of MSCs (which in turn induces spheroid formation) recovers the cell communication and provides a concertation gradient of external factors depending on the location (core to marginal region) as observed in vivo, MSC spheroid exhibits superior viability and self-renew capacity with enhanced differentiation potential compared to 2D cells [132]. Moreover, 3D cells tend to produce a higher level of therapeutic paracrine molecules than 2D cells; indeed, the secretion of anti-inflammatory factors such as TSG-6, PGE2, Stanniocalcin-1, Leukemia inhibitory factor (LIF) and TGF-β is significantly stimulated upon cell aggregation [133]. collectively, the immunomodulatory efficacy of 3D cells is generally superior to that of 2D cells. Interestingly, 3D assembly of MSCs drives transcriptome change with activation of immune responsive pathways including chemokines and IL-1 signaling and these pro-inflammatory agents within the spheroid microenvironment can further prime MSCs to adopt anti-inflammatory properties [134,135,136]. Thomas et al. found that both neutralization of IL-1 activity and prevention of pro-IL-1 cleavage with caspase inhibitor significantly decreased the anti-inflammatory effect of 3D cells against macrophage activation [135]. In addition, treatment of γ-secretase inhibitor during the MSC spheroid culture suppressed the secretion of PGE2 in a dose-dependent manner, indicating the involvement of contact-dependent Notch signaling in this phenomenon.

The anti-inflammatory property of MSC aggregates has been demonstrated in vitro and in vivo (Table 3). Several works have been indicated that both MSC spheroid itself and its conditioned media stimulate the macrophage polarization into M2 phenotype via PGE2 and its receptor EP4-mediated regulation [133,135,137]. In addition, intraperitoneally transfused MSC spheroid could reduce the total volume and protein content of ascites in the zymogen-induced murine peritonitis model with improved inflammatory signs [136]. The intraportal injection of 3D MSCs rescued the macrophagic M1/M2 imbalance by inducing M2 differentiation in galactosamine/LPS (GalN/LPS)-mediated hepatic injury mouse [138]. Moreover, the immunomodulatory function of MSC spheroid can be further enhanced by combining priming strategies as described in Section 4.1.1. For instance, 3D MSCs given pre-treatment of IL-1α and β could reduce the TNF-α secretion level in LPS-activated murine microglia cell BV2 to a greater extend to naïve 3D cells [139]. In addition, priming of MSC spheroid with IFN-γ and TNF-α led to a great increment in the production of MSC-derived immunomodulatory factors such as PGE2 and kynurenine, resulting in the suppression of the pro-inflammatory response of macrophage [140].

At present, the hanging-drop culture, forced aggregation technique and culture on the low-attachment micro-well plate are the simplest as well as the most widely used 3D culture techniques in the basic research field; however, these methods are labor-intensive and often lead to low yields of spheroids. Therefore, several advanced techniques such as utilizing biocompatible scaffold, microbioreactor and robotics-based 3D printing have been developed to produce a sufficient number of homogenous spheroid [141]. In addition, different culture conditions such as media type, composition and the presence of serum can significantly influence not only cell yield but also the immunophenotype of MSC spheroid [137,140]. Since cells residing at the core have to be exposed to hypoxia and mechanical stress, the optimal cell packing density and average diameter of aggregates must be determined during spheroid generation. Interestingly, Shobha et al. generated heterospheroid by combining 3D MSCs with anti-oxidative agent quercetin to potentiate the viability of MSCs. Interestingly, the delivery of quercetin could prevent apoptosis of center-positioned cells and thus improved their therapeutic capacity against DSS-induced colitis model [142]. In addition, Murphy et al. have designed an analysis platform to estimate how three culture variables, which are cell count per spheroid, oxygen concentration and immune mediators, can affect the functionality of MSC spheroid. Using this approach, the authors specified the best combination of culture conditions to generate the most potent BM-MSC aggregates in terms of secretion of PGE2 and VEGF [143], providing some insights on how to optimize the MSC spheroid culture procedure for the clinical translation.

#### 4.2.2. Encapsulation or Embedding of MSCs with Biocompatible Agents

For the reconstitution of MSC niche-mimicking environment in vitro, various bioactive materials can be utilized as the encapsulating hydrogel as well as the biomimetic scaffolds [144]. They can offer MSCs with stable “tissue-like” microenvironment with sufficient cell-to-ECM interactions, which enables MSCs to maintain their therapeutic potency in vivo as observed in vitro. Both natural and synthetic biomaterials can be applied for the 3D culture of MSCs. The most widely-used natural materials include alginate, hyaluronic acid (HA), chitosan, collagen, gelatin and fibrin, while poly ethylene glycol (PEG) and poly-(l-lactic acid), poly(lactic-co-glycolic acid) (PLGA) represent the synthetic materials at present. Each material can be cross-linked together to synthesize new copolymer structures (i.e., PEG-PLGA).

The positive impact of biomaterial-based 3D structure on MSC-mediated immunomodulation has been demonstrated over the past decade (Table 4). For instance, alginate microencapsulation can induce immune-phenotype of MSCs towards an anti-inflammatory direction. It has been reported that alginate-MSC hydrogel induced macrophage polarization towards anti-inflammatory M2 type differentiation and prevented PBMC proliferation significantly, while it did not elicit DC maturation and activation [145,146]. Zanotti et al. also studied the immune-regulatory capacity of alginate encapsulated MSCs in murine GvHD model and found that intravenously injected MSCs could reduce the proliferation of both CD4+ and CD8+ T cells and ameliorated the infiltration of immune cells in the liver, leading to the overall enhancement in the clinical score and survival of GvHD mouse [147]. In a spinal cord injury model, alginate-MSC hydrogel reduced the neuro-inflammatory signs by preventing the pro-inflammatory reactive microgliosis and astrocytosis [146]. The neuroprotective impact of encapsulated MSCs has also been addressed on LPS-treated organotypic hippocampal slice, where MSC-derived PGE2 was involved in the reduction of TNF-α level [148]. Similar to encapsulated MSCs, MSCs cultured within the biomaterial 3D scaffold exerted a superior anti-inflammatory impact on innate- and adaptive immune cells compared to 2D cultured MSCs [149,150].

It has been noted that biomaterials modulate the MSC behavior in terms of differentiation, proliferation, mobility (retention at the injected site or homing to other targets) and paracrine activity depending on the combination of physical parameters such as stiffness, degradability, polarity and porosity. A recent study has investigated how the rigidity of encapsulating material affects the transcriptome of MSCs cultured in alginate hydrogels at different stiffness by bulk sequencing [151]. The main signatures of differentially expressed genes were involved in cell-substrate adhesion, proteolysis and developmental pathway, along with immune-related processes such as IL-1 signaling. Intriguingly, an increase in alginate stiffness led to an up-regulation of the NF-kB subunit p65 and IDO expression in MSCs, implying that the activity of central immune mediators including NF-kB and CREB signaling could be regulated by the substrate stiffness. In another study, three HAs with different molecular weight (1.6 MDa, 150 kDa or 7.5 kDa) was applied for microencapsulation of MSCs and their immune-related activity was evaluated with PBMC, T cells and monocytes to study the influence of the molecular weight of biomaterials on MSC functionality [152]. It has been noted that although the higher molecular weight of HA (hHA) itself led to an unexpected slight increase in PBMC proliferation, application of hHA could enhance the immunomodulatory capacity of MSCs in terms of induction of IL-10 secretory Th cells and M2 macrophages. Meanwhile, the fibrous topography of scaffolds is another important determinant for MSC regulation [153,154]. To investigate the contribution of nanofiber orientation in the scaffold to the paracrine function of MSCs, MSCs were cultured on the 2D plate or 3D scaffolds that consist of electrospun fibers with random, mesh-like or aligned structures and their secretory profiles were evaluated [153]. Interestingly, differences in fiber arrangement of 3D scaffold can significantly affect the paracrine activity of MSCs and conditioned media (CM) obtained from MSCs on mesh-like structure (MSC-MEF CM) displayed the most potent anti-inflammatory roles in macrophage inhibition. Moreover, upon the topical application in the skin defect model, MSC-MEF CM accelerated the wound healing process via recruiting the pro-regenerative CD206 + M2 macrophages into the wound bed. In another report by Wan and colleagues, the authors compared the immunophenotype of MSCs cultured on random or aligned fibrous scaffold [154]. They found that aligned fiber structure was ideal to upregulate the immunoregulatory capacity of MSCs than the randomly assembled scaffold. Mechanistically, aligned microenvironment-mediated mechanotransduction induced the stimulation of the Yes-associated protein (YAP) pathway as well as focal adhesion kinase (FAK)-ERK1/2 signaling cascade in MSCs, resulting in enhanced immunomodulatory properties. Collectively, these observations emphasize the importance of the hydrogel fabrication method in the regulation of the MSC functions.

Meanwhile, MSC encapsulation technique can overcome the several limitations of the conventional single cell- or spheroid injection [158]; first, biomaterials function as a physical barrier of MSCs against harsh environmental conditions such as damaged tissue-derived cytotoxic signals and host immune responses, leading to the prolonged survival of MSCs in vivo. Moreover, the natural ECM-mimicking domain can be tethered into the substrate in an attempt to enhance cell adhesion and viability. For instance, PPFLMLLKGSTR peptide-bearing HA scaffold significantly improved the MSC viability than naïve HA, contributing to the effective nerve regeneration with decreased astrocytic activation upon MSC-scaffold implantation in spinal cord injury model [155]. The fibronectin-derived Arg-Gly-Asp (RGD) motif is another commonly used peptide for this purpose [145]. In addition, encapsulated MSCs can be primed by tethering the pro-inflammatory agent such as IFN-γ into hydrogel [156] as described in Section 4.1.1.

A recent work by Mao et al. has suggested an advanced strategy for the practical usage of the current technique [157]. Using a microfluidic device, a single cell can be encapsulated into a multi-layered microgel composed of alginate-poly-D-lysine (PDL)-alginate (APA) coating. These microgels exerted resistance to the cytotoxic damage caused by the repeated freeze-thawing cycle. The enclosed MSCs could proliferate normally, generating a clonally identical multicellular structure (MAPA). In the form of MAPA, MSCs produced a higher level of immunomodulatory paracrine factors including IL-10, Cox-2, TGF-β and TSG-6 than control cells. Upon in vivo administration via the intravenous route, both naïve single cells and MAPA are predominantly trapped in the lung then single cells were disappeared rapidly as reported previously [159]; on the contrary, MAPA exhibited a significantly prolonged half-life and higher residence capacity than bare cells without causing any host pathological responses. In addition, licensing factors such as TNFα and IFN-γ could further reinforce the therapeutic immunophenotype of MAPA; indeed, primed MAPA reduced host immune rejection responses and, in turn, supported the engraftment of allogeneic BM transplant in the mouse model to a great extent to unprimed control. Therefore, the application of this specialized microencapsulation technique with a programmable multi-layered structure resulted in an overall improvement in the immunomodulatory capacity of MSCs.

## 5. Conclusions and Future Perspectives

During the last decade, MSCs have been suggested as promising therapeutics for the treatment of various immune disorders and a large body of preclinical and clinical studies have been reported. More recently, as summarized in this review, researchers have developed several bioengineering technologies to generate highly efficient MSCs to overcome previously reported limitations of MSC application mainly mediated by non-uniform functional potency and rapid clearance after transplantation. For the clinical application of these latest technologies, future studies should intensively focus on the verification of the safety of manipulated cells, as well as the development of the standard platform for the quality control of clinically potent cells.

## Figures and Tables

**Table 2:** The immunomodulatory impact of genetically modified MSCs on animal models.

Target Factor	Engineering Method	Cell Source/Route of Injection	Animal Model/Immune-Related In Vivo Effect	References
IL-10	Lentivirus	mBM-MSC Intracerebral	TBI model Astrosytosis & Microgliosis ↓	[99]
IL-10	Lentivirus	hAD-MSC Intraperitoneal	EAE model/	[98]
			Treg ↑, Th17 ↓, DC maturation ↓	
IL-10	AAV	hBM-MSC Intravenous	MCAO model Microgliosis ↓, Pro-inflammtory cytokine ↓	[97]
IL-10	Lentivirus	Dark-Aguti MSC Intravenous	acute liver allograft rejection model/ Treg ↑, Th17 ↓	[100]
IL-10	Retrovirus	hBM-MSC Intravenous	Lung Ischemia–Reperfusion Injury/ Granulocyte, CD4+ & CD8+ T cells ↓ Treg ↑ in BAL	[102]
IL-10	Retrovirus	mBM-MSC Intravenous	LPS-Induced ALI model/ IL-10 producing CD4+ &CD8+ T cells, B cells ↑ TNF-α ↓in BAL	[101]
IL-4	Lentivirus	hAD-MSC Intraperitoneal	EAE model/ Th1/Th17 ↓, Th2 response ↑	[103]
IL-4	Lentivirus	mAD-MSC (single cells and spheroid) Intra-articular	Osteoarthritis model/ NO mediated damage ↓	[104]
GM-CSF	Lentivirus	mBM-MSC Intraperitoneal	ECDC model/ CD11b+GR-1+ MDSC & Treg mobilization ↑, Th17 ↓	[105]
IFN-γ	Lentivirus	mAD-MSC Intravenous	EAE model/ Treg ↑, CD3+ & CD4+ T cells ↓	[106]
IL-1Ra	Lentivirus	hAF-MSC	FHF model/	[107]
		Portal vein injection	infiltration of mononuclear cells ↓	
IL-37	Lentivirus	mBM-MSC Intravenous	MRL/lpr mice (model of SLE)/ B220+, CD3+, CD4+, CD8+, CD11b+, B220+CD3+, CD138+IgG+ and CD4+IL17+ cells ↓Treg ↑	[108]
sST2	Lentivirus	hAD-MSC Intravenous	LPS-Induced ALI model/ Pro- IL-33, TLR4, IL-1β and IFN-γ ↓IL-10 ↑	[109]
IL-10	CRISPR/Cas9 (dCas-SAM system)	mBM-MSC/ Intramyocardial	myocardial infarction in diabetes model/ CD68+ CD11b+ cells ↓ in the heart Pro-inflammatory cytokine ↓	[110]
miR-223	Lentivirus	mBM-MSC/ Intraperitoneal exosome treatment	experimental autoimmune hepatitis model/ NLRP3 inflammasome activation ↓ IL-1β, TNF-α, IL-17 ↓	[111]
miR-181a	Lentivirus	hUCB-MSC/ Intramyocardial exosome treatment	myocardial ischemia-reperfusion injury model/ Treg ↑ in the heart	[112]
miR-181-5p	Plasmid transfection	mAD-MSC/ Intrasplenic exosome treatment	Liver fibrosis model/ TNF-α, IL-6, IL-17 ↓ in the liver	[113]
miR-30d-5p	Plasmid transfection	ratAD-MSC/ Intravenous exosome treatment	Ischemic stroke model/ Microglial autophagy ↓ M1 polarization ↓	[114]
Angiopoietin1	Plasmid electroporation	mBM-MSC/ Intravenous	LPS-Induced ALI model/ TNF-α, IL-6, IL-8, Cxcl2 ↓ in the lung	[115]
SOD3	Lentivirus	hUCB-MSC/ Subcutaneous	Imiquimod-induced psoriasis-like model/ lymphocyte, DC, neutrophil infiltration ↓ in the skin	[116]
SOD3	Lentivirus	hUCB-MSC/ Subcutaneous MSCs or exosome treatment	atopic dermatitis model/ lymphocyte and mast cell infiltration ↓	[117]

IL; interleukin, m; mouse, h; human, BM; bone marrow, Treg; regulatory T cell, Th; helper T cell, AD; adipose tissue-derived, TBI; traumatic brain injury, EAE; experimental autoimmune encephalomyelitis, MCAO; middle cerebral artery occlusion, BAL; bronchoalveolar lavage, LPS; lipopolysaccharide, ALI; acute lung injury, NO; nitric oxide, GM-SCF; granulocyte-colony stimulating factor, ECDC; experimental Chagas disease cardiomyopathy, MDSC; myeloid-derived suppressor cell, IFN; interferon, FHF; fulminant hepatic failure, AF; amniotic fluid, SLE; Systemic Lupus Erythematosus, sST2; soluble IL-33/IL-1 receptor–like–1, dCas-SAM; de-activated Cas-Synergistic activation mediator, UCB; umbilical cord blood, DC; dendritic cell, SOD3; superoxide dismutase 3.

**Table 3:** Influence of 3D assembly on MSC-mediated immunoregulatory functions.

Strategy	Method/ Biomaterial	Cell Source	In Vitro/In Vivo Immunomodulatory Effect	References
3D spheroid	Hanging drop	hBM-MSC	(in vitro) PGE2 ↑, M2 ↑	[133,137]
3D spheroid	Hanging drop	hBM-MSC	(in vitro) Self-activation of IL1 pathway PGE2 ↑, M ↓, M2 ↑	[135]
3D spheroid	Hanging drop	hBM-MSC	(in vitro) TSG-6 ↑, STC-1 ↑, LIF ↑ (in vivo) M1 ↓ peritonitis model	[136]
3D spheroid	Hanging drop	hAD-MSC	(in vitro) PGE2 ↑, M1 ↓, M2 ↑ (in vivo) CD11b+F4/80+ cell ↓, M1 ↓, M2 ↑ in FHF model	[138]
3D spheroid + IL-1αβ priming	Hanging drop	hBM-MSC	(in vitro) priming enhanced TNF-α ↓ in LPS-treated BV2 cell	[139]
3D spheroid + TNF-α, IFN-γ priming	Forced aggregation	hAD-MSC	(in vitro) priming enhanced M1 ↓	[140]

**Table 4:** The biomaterial-based structural modification of MSCs to boost immunomodulatory property.

Strategy	Method/ Biomaterial	Cell Source	In Vitro/In Vivo Immunomodulatory Effect	References
Encapsulation	Alginate	mBM-MSC	(in vivo) CD4+, CD8+and CD11c+ cells ↓in Murine GvHD model	[147]
Encapsulation	Alginate-PLL	hBM-MSC	(in vitro) M1 ↓, M2 ↑ (in vivo) recruitment of M2 in the lesion of SCI model	[146]
Encapsulation	Alginate with RGD motif	hAD-MSC	(in vitro) PBMC proliferation ↓	[145]
Encapsulation	Alginate-PLL	hBM-MSC	(ex vivo) PGE2 ↑, TNF-α ↓ in hippocampal slice culture	[148]
3D scaffold embedding	Collagen, chitosan, PLGA	hUCB-MSC	(in vitro) CD73 ↓ in 3D MSCs T cell proliferation ↓	[150]
3D scaffold embedding	HA-gelatin	hBM, AD, VF MSC	(in vitro) CD16 ↓ in monocyte co-cultured with 3D MSC	[149]
3D scaffold embedding	Alginate with different stiffness	mMSC	(in vitro) NF-kB subunit p65 and IDO ↑ in MSCs cultured within stiff gel	[151]
3D scaffold embedding	HA with various MW	hBM-MSC	(in vitro) high MW HA-derived MSC further increased M2 ↑	[152]
3D scaffold embedding	PCL EF with various orientation	Rat AD-MSC	(in vitro) MSCs cultured on mesh-like scaffold were most potent in M1 ↓, M2 ↑	[153]
3D scaffold embedding	PLLA EF with various orientation	hAD-MSC	(in vitro) PGE2 ↑, TSG6 ↑in MSCs cultured on aligned scaffold	[154]
3D scaffold embedding	HA with Adhesive motif	Rat BM-MSC	(in vivo) CD68+ cell ↓, glial scar ↓in spinal cord transection model	[155]
3D scaffold embedding	PEG hydrogel with IFN-γ functionalization	hBM-MSC	(in vitro) increase in MCP-1, M-CSF, CXCL9, CXCL10 and CCL8 in MSCs cultured within PEG-INF-γ scaffold	[156]
Encapsulation + TNF-α, IFN-γ priming	APA construct	hBM-MSC mBM-MSC	(in vitro) increase in IL-10, IL-6, Cox-2, TGF-β and TSG-6 in the form of MAPA	[157]

PLL; Poly-L-Lysine, SCI; spinal cord injury, RGD; Arg-Gly-Asp, PLGA; poly(lactic-co-glycolic acid), HA; hyaluronic acid, MW; molecular weight, PCL EF; polycaprolactone electrospun fiber, PEG; poly(ethylene glycol), APA; alginate to form alginate–PDL–alginate, MAPA; multicellular APA.

## References

[1] Le Blanc K., Mougiakakos D. (2012). Multipotent mesenchymal stromal cells and the innate immune system. Nat. Rev. Immunol..

[2] Wang Y., Chen X., Cao W., Shi Y. (2014). Plasticity of mesenchymal stem cells in immunomodulation: Pathological and therapeutic implications. Nat. Immunol..

[3] Wang M., Yuan Q., Xie L. (2018). Mesenchymal Stem Cell-Based Immunomodulation: Properties and Clinical Application. Stem Cells Int..

[4] Lee J.W., Gupta N., Serikov V., A Matthay M. (2009). Potential application of mesenchymal stem cells in acute lung injury. Expert Opin. Biol. Ther..

[5] Aggarwal S., Pittenger M.F. (2005). Human mesenchymal stem cells modulate allogeneic immune cell responses. Blood.

[6] Meisel R., Zibert A., Laryea M., Goöbel U., Daäubener W., Dilloo D. (2004). Human bone marrow stromal cells inhibit allogeneic T-cell responses by indoleamine 2,3-dioxygenase–mediated tryptophan degradation. Blood.

[7] Lepelletier Y., Lecourt S., Renand A., Arnulf B., Vanneaux V., Fermand J.-P., Menasché P., Domet T., Marolleau J.-P., Hermine O. (2010). Galectin-1 and Semaphorin-3A Are Two Soluble Factors Conferring T-Cell Immunosuppression to Bone Marrow Mesenchymal Stem Cell. Stem Cells Dev..

[8] RalfHass R., Kasper C., Böhm S., Jacobs T.C.R. (2011). Different populations and sources of human mesenchymal stem cells (MSC): A comparison of adult and neonatal tissue-derived MSC. Cell Commun. Signal..

[9] De Wolf C., van de Bovenkamp M., Hoefnagel M. (2017). Regulatory perspective on in vitro potency assays for human mesenchymal stromal cells used in immunotherapy. Cytotherapy.

[10] Trivedi A., Miyazawa B., Gibb S., Valanoski K., Vivona L., Lin M., Potter D., Stone M., Norris P.J., Murphy J. (2019). Bone marrow donor selection and characterization of MSCs is critical for pre-clinical and clinical cell dose production. J. Transl. Med..

[11] Levy O., Kuai R., Siren E.M.J., Bhere D., Milton Y., Nissar N., De Biasio M., Heinelt M., Reeve B., Abdi R. (2020). Shattering barriers toward clinically meaningful MSC therapies. Sci. Adv..

[12] Glass C.K., Natoli G. (2016). Molecular control of activation and priming in macrophages. Nat. Immunol..

[13] Lucas T., Waisman A., Ranjan R., Roes J., Krieg T., Müller W., Roers A., Eming S.A. (2010). Differential Roles of Macrophages in Diverse Phases of Skin Repair. J. Immunol..

[14] Vasandan A.B., Jahnavi S., Shashank C., Prasad P., Kumar A., Prasanna S.J. (2016). Human Mesenchymal stem cells program macrophage plasticity by altering their metabolic status via a PGE2-dependent mechanism. Sci. Rep..

[15] Liu F., Qiu H., Xue M., Zhang S., Zhang X., Xu J., Chen J., Yang Y., Xie J. (2019). MSC-secreted TGF-β regulates lipopolysaccharide-stimulated macrophage M2-like polarization via the Akt/FoxO1 pathway. Stem Cell Res. Ther..

[16] Wculek S.K., Cueto F.J., Mujal A.M., Melero I., Krummel M.F., Sancho D. (2020). Dendritic cells in cancer immunology and immunotherapy. Nat. Rev. Immunol..

[17] Jiang X.-X., Zhang Y., Liu B., Zhang S.-X., Wu Y., Yu X.-D., Mao N. (2005). Human mesenchymal stem cells inhibit differentiation and function of monocyte-derived dendritic cells. Blood.

[18] Favaro E., Carpanetto A., Caorsi C., Giovarelli M., Angelini C., Cavallo-Perin P., Tetta C., Camussi G., Zanone M.M. (2016). Human mesenchymal stem cells and derived extracellular vesicles induce regulatory dendritic cells in type 1 diabetic patients. Diabetologia.

[19] Lu Z., Chang W., Meng S., Xu X., Xie J., Guo F., Yang Y., Qiu H., Liu L. (2019). Mesenchymal stem cells induce dendritic cell immune tolerance via paracrine hepatocyte growth factor to alleviate acute lung injury. Stem Cell Res. Ther..

[20] Abel A.M., Yang C., Thakar M.S., Malarkannan S. (2018). Natural Killer Cells: Development, Maturation, and Clinical Utilization. Front. Immunol..

[21] Fathman J.W., Bhattacharya D., Inlay M.A., Seita J., Karsunky H., Weissman I.L. (2011). Identification of the earliest natural killer cell–committed progenitor in murine bone marrow. Blood.

[22] Selmani Z., Naji A., Zidi I., Favier B., Gaiffe E., Obert L., Borg C., Saas P., Tiberghien P., Rouas-Freiss N. (2008). Human Leukocyte Antigen-G5 Secretion by Human Mesenchymal Stem Cells Is Required to Suppress T Lymphocyte and Natural Killer Function and to Induce CD4+CD25highFOXP3+Regulatory T Cells. Stem Cells.

[23] Spaggiari G.M., Capobianco A., Abdelrazik H., Becchetti F., Mingari M.C., Moretta L. (2008). Mesenchymal stem cells inhibit natural killer–cell proliferation, cytotoxicity, and cytokine production: Role of indoleamine 2,3-dioxygenase and prostaglandin E2. Blood.

[24] Najar M., Fayyad-Kazan M., Meuleman N., Bron D., Fayyad-Kazan H., Lagneaux L. (2018). Immunomodulatory effects of foreskin mesenchymal stromal cells on natural killer cells. J. Cell. Physiol..

[25] Malech H.L., DeLeo F.R., Quinn M.T. (2014). The Role of Neutrophils in the Immune System: An Overview. Methods Mol. Biol..

[26] Li Y., Wang W., Yang F., Xu Y., Feng C., Zhao Y. (2019). The regulatory roles of neutrophils in adaptive immunity. Cell Commun. Signal..

[27] Raffaghello L., Bianchi G., Bertolotto M., Montecucco F., Busca A., Dallegri F., Ottonello L., Pistoia V. (2008). Human Mesenchymal Stem Cells Inhibit Neutrophil Apoptosis: A Model for Neutrophil Preservation in the Bone Marrow Niche. Stem Cells.

[28] Jiang D., Muschhammer J., Qi Y., Kügler A., De Vries J.C., Saffarzadeh M., Sindrilaru A., Beken S.V., Wlaschek M., Kluth M.A. (2016). Suppression of Neutrophil-Mediated Tissue Damage-A Novel Skill of Mesenchymal Stem Cells. Stem Cells.

[29] Glennie S., Soeiro I., Dyson P.J., Lam E.W.-F., Dazzi F., Lutsiak M.E.C., Semnani R.T., De Pascalis R., Kashmiri S.V.S., Schlom J. (2005). Bone marrow mesenchymal stem cells induce division arrest anergy of activated T cells. Blood.

[30] Davies L.C., Heldring N., Kadri N., Le Blanc K. (2017). Mesenchymal Stromal Cell Secretion of Programmed Death-1 Ligands Regulates T Cell Mediated Immunosuppression. Stem Cells.

[31] Jiang W., Xu J. (2019). Immune modulation by mesenchymal stem cells. Cell Prolif..

[32] Ghannam S., Pène J., Torcy-Moquet G., Jorgensen C., Yssel H. (2010). Mesenchymal Stem Cells Inhibit Human Th17 Cell Differentiation and Function and Induce a T Regulatory Cell Phenotype. J. Immunol..

[33] Rashedi I., Gómez-Aristizábal A., Wang X.-H., Viswanathan S., Keating A. (2017). TLR3 or TLR4 Activation Enhances Mesenchymal Stromal Cell-Mediated Treg Induction via Notch Signaling. Stem Cells.

[34] Negi N., Griffin M.D. (2020). Effects of mesenchymal stromal cells on regulatory T cells: Current understanding and clinical relevance. Stem Cells.

[35] Luz-Crawford P., Kurte M., Bravo-Alegría J., Contreras R., Nova-Lamperti E., Tejedor G., Noël D., Jorgensen C., Figueroa F., Djouad F. (2013). Mesenchymal stem cells generate a CD4+CD25+Foxp3+ regulatory T cell population during the differentiation process of Th1 and Th17 cells. Stem Cell Res. Ther..

[36] Franquesa M., Hoogduijn M.J., Bestard O., Grinyó J.M. (2012). Immunomodulatory Effect of Mesenchymal Stem Cells on B Cells. Front. Immunol..

[37] Rafei M., Hsieh J., Fortier S., Li M., Yuan S., Birman E., Forner K., Boivin M.-N., Doody K., Tremblay M. (2008). Mesenchymal stromal cell–derived CCL2 suppresses plasma cell immunoglobulin production via STAT3 inactivation and PAX5 induction. Blood.

[38] Schena F., Gambini C., Gregorio A., Mosconi M., Reverberi D., Gattorno M., Casazza S., Uccelli A., Moretta L., Martini A. (2010). Interferon-γ-dependent inhibition of B cell activation by bone marrow-derived mesenchymal stem cells in a murine model of systemic lupus erythematosus. Arthritis Rheum..

[39] Luz-Crawford P., Djouad F., Toupet K., Bony C., Franquesa M., Hoogduijn M.J., Jorgensen C., Noël D. (2015). Mesenchymal Stem Cell-Derived Interleukin 1 Receptor Antagonist Promotes Macrophage Polarization and Inhibits B Cell Differentiation. Stem Cells.

[40] Franquesa M., Mensah F.K., Huizinga R., Strini T., Boon L., Lombardo E., Delarosa O., Laman J.D., Grinyó J.M., Weimar W. (2015). Human Adipose Tissue-Derived Mesenchymal Stem Cells Abrogate Plasmablast Formation and Induce Regulatory B Cells Independently of T Helper Cells. Stem Cells.

[41] Gebler A., Zabel O., Seliger B. (2012). The immunomodulatory capacity of mesenchymal stem cells. Trends Mol. Med..

[42] Kalinski P. (2011). Regulation of Immune Responses by Prostaglandin E2. J. Immunol..

[43] Le Blanc K., Davies L.C. (2015). Mesenchymal stromal cells and the innate immune response. Immunol. Lett..

[44] Wang D., Huang S., Yuan X., Liang J., Xu R., Yao G., Feng X., Sun L. (2015). The regulation of the Treg/Th17 balance by mesenchymal stem cells in human systemic lupus erythematosus. Cell. Mol. Immunol..

[45] Gazdic M., Markovic B.S., Vucicevic L., Nikolic T., Djonov V., Arsenijevic N., Trajkovic V., Lukic M.L., Volarevic V. (2017). Mesenchymal stem cells protect from acute liver injury by attenuating hepatotoxicity of liver natural killer T cells in an inducible nitric oxide synthase- and indoleamine 2,3-dioxygenase-dependent manner. J. Tissue Eng. Regen. Med..

[46] Sato K., Ozaki K., Oh I., Meguro A., Hatanaka K., Nagai T., Muroi K., Ozawa K. (2006). Nitric oxide plays a critical role in suppression of T-cell proliferation by mesenchymal stem cells. Blood.

[47] Crop M., Baan C., Korevaar S., Ijzermans J., Pescatori M., Stubbs A., Van Ijcken W., Dahlke M., Eggenhofer E., Weimar W. (2010). Inflammatory conditions affect gene expression and function of human adipose tissue-derived mesenchymal stem cells. Clin. Exp. Immunol..

[48] Ren G., Zhang L., Zhao X., Xu G., Zhang Y., Roberts A.I., Zhao R.C., Shi Y. (2008). Mesenchymal Stem Cell-Mediated Immunosuppression Occurs via Concerted Action of Chemokines and Nitric Oxide. Cell Stem Cell.

[49] Kabat M., Bobkov I., Kumar S., Grumet M. (2020). Trends in mesenchymal stem cell clinical trials 2004-2018: Is efficacy optimal in a narrow dose range?. Stem Cells Transl. Med..

[50] Ullah M., Liu D.D., Thakor A.S. (2019). Mesenchymal Stromal Cell Homing: Mechanisms and Strategies for Improvement. iScience.

[51] Moll G., Ankrum J.A., Kamhieh-Milz J., Bieback K., Ringdén O., Volk H.-D., Geissler S., Reinke P. (2019). Intravascular Mesenchymal Stromal/Stem Cell Therapy Product Diversification: Time for New Clinical Guidelines. Trends Mol. Med..

[52] (2021). Early clinical, histological, and immunohistochemical findings in suspected acute graft-versus-host disease and their association with patient outcomes. Pediatr. Dermatol..

[53] Fletcher J.M., Lalor S.J., Sweeney C.M., Tubridy N., Mills K.H.G. (2010). T cells in multiple sclerosis and experimental autoimmune encephalomyelitis. Clin. Exp. Immunol..

[54] Venema W.T.C.U., Voskuil M.D., Dijkstra G., Weersma R.K., Festen E.A.M. (2016). The genetic background of inflammatory bowel disease: From correlation to causality. J. Pathol..

[55] Duan L., Huang H., Cao X., Zhao X., Zhou M., Chen S., Wang C., Han Z., Han Z.-C., Guo Z. (2020). Extracellular vesicles derived from human placental mesenchymal stem cells alleviate experimental colitis in mice by inhibiting inflammation and oxidative stress. Int. J. Mol. Med..

[56] Holan V., Hermankova B., Bohacova P., Kossl J., Chudickova M., Hajkova M., Krulova M., Zajicova A., Javorkova E. (2016). Distinct Immunoregulatory Mechanisms in Mesenchymal Stem Cells: Role of the Cytokine Environment. Stem Cell Rev. Rep..

[57] De Miguel M.P., Fuentes-Julian S., Blazquez-Martinez A., Pascual C.Y., Aller M.A., Arias J., Arnalich-Montiel F. (2012). Immunosuppressive Properties of Mesenchymal Stem Cells: Advances and Applications. Curr. Mol. Med..

[58] Kim H., Shin T., Lee B., Yu K., Seo Y., Lee S., Seo M., Hong I., Choi S.W., Seo K. (2013). Human Umbilical Cord Blood Mesenchymal Stem Cells Reduce Colitis in Mice by Activating NOD2 Signaling to COX2. Gastroenterology.

[59] Chen Q.-Q., Yan L., Wang C.-Z., Wang W.-H., Shi H., Su B.-B., Zeng Q.-H., Du H.-T., Wan J. (2013). Mesenchymal stem cells alleviate TNBS-induced colitis by modulating inflammatory and autoimmune responses. World J. Gastroenterol..

[60] Gile J.J., Sara J.D.S., Mueller M.R. (2021). Systemic lupus erythematosus multiorgan flare with quiescent serologic markers. BMJ Case Rep..

[61] Islam A., Khandker S.S., Alam S.S., Kotyla P., Hassan R. (2019). Vitamin D status in patients with systemic lupus erythematosus (SLE): A systematic review and meta-analysis. Autoimmun. Rev..

[62] Boberg E., Von Bahr L., Afram G., Lindström C., Ljungman P., Heldring N., Petzelbauer P., Legert K.G., Kadri N., Le Blanc K. (2020). Treatment of chronic GvHD with mesenchymal stromal cells induces durable responses: A phase II study. Stem Cells Transl. Med..

[63] Dhere T., Copland I., Garcia M., Chiang K.Y., Chinnadurai R., Prasad M., Galipeau J., Kugathasan S. (2016). Randomised clinical trial: Safety of autologous and metabolically fit bone marrow mesenchymal stromal cells in medically refractory Crohn’s disease-a phase 1 trial with three doses. Aliment. Pharmacol. Ther..

[64] Ciccocioppo R., Bernardo M.E., Sgarella A., Maccario R., Avanzini M.A., Ubezio C., Minelli A., Alvisi C., Vanoli A., Calliada F. (2011). Autologous bone marrow-derived mesenchymal stromal cells in the treatment of fistulising Crohn’s disease. Gut.

[65] Llufriu S., Sepulveda M., Blanco Y., Marin P., Moreno B., Berenguer J., Gabilondo I., Martinez-Heras E., Sola-Valls N., Arnaiz J. (2014). Randomized Placebo-Controlled Phase II Trial of Autologous Mesenchymal Stem Cells in Multiple Sclerosis. PLoS ONE.

[66] Lee B.-C., Kang K.-S. (2020). Functional enhancement strategies for immunomodulation of mesenchymal stem cells and their therapeutic application. Stem Cell Res. Ther..

[67] Seo Y., Shin T.-H., Kim H.-S. (2019). Current Strategies to Enhance Adipose Stem Cell Function: An Update. Int. J. Mol. Sci..

[68] Yan L., Zheng D., Xu R.-H. (2018). Critical Role of Tumor Necrosis Factor Signaling in Mesenchymal Stem Cell-Based Therapy for Autoimmune and Inflammatory Diseases. Front. Immunol..

[69] Nc N.D.C.N., Mizukami A., Caliári-Oliveira C., Cominal J.G., Rocha J.L.M., Covas D.T., Swiech K., Malmegrim K.C.R. (2019). Priming approaches to improve the efficacy of mesenchymal stromal cell-based therapies. Stem Cell Res. Ther..

[70] Boland L., Burand A.J., Brown A.J., Boyt D., Lira V.A., Ankrum J.A. (2018). IFN-γ and TNF-α Pre-licensing Protects Mesenchymal Stromal Cells from the Pro-inflammatory Effects of Palmitate. Mol. Ther..

[71] Carrero R., Cerrada I., Lledó E., Dopazo J., García-García F., Rubio M.-P., Trigueros C., Dorronsoro A., Ruiz-Sauri A., Montero J.A. (2012). IL1β Induces Mesenchymal Stem Cells Migration and Leucocyte Chemotaxis Through NF-κB. Stem Cell Rev. Rep..

[72] Vigo T., Procaccini C., Ferrara G., Baranzini S., Oksenberg J.R., Matarese G., Diaspro A., De Rosbo N.K., Uccelli A. (2017). IFN-γ orchestrates mesenchymal stem cell plasticity through the signal transducer and activator of transcription 1 and 3 and mammalian target of rapamycin pathways. J. Allergy Clin. Immunol..

[73] Kim D.S., Jang I.K., Lee M.W., Ko Y.J., Lee D.-H., Lee J.W., Sung K.W., Koo H.H., Yoo K.H. (2018). Enhanced Immunosuppressive Properties of Human Mesenchymal Stem Cells Primed by Interferon-γ. EBioMedicine.

[74] Klinker M.W., Marklein R.A., Surdo J.L.L., Wei C.-H., Bauer S.R. (2017). Morphological features of IFN-γ–stimulated mesenchymal stromal cells predict overall immunosuppressive capacity. Proc. Natl. Acad. Sci. USA.

[75] Lee B.-C., Kim J.-J., Lee J.Y., Kang I., Shin N., Lee S.-E., Choi S.W., Cho J.-Y., Kim H.-S., Kang K.-S. (2019). Disease-specific primed human adult stem cells effectively ameliorate experimental atopic dermatitis in mice. Theranostics.

[76] Duijvestein M., Wildenberg M.E., Welling M.M., Hennink S., Molendijk I., Van Zuylen V.L., Bosse T., Vos A.C.W., De Jonge-Muller E.S.M., Roelofs H. (2011). Pretreatment with Interferon-γ Enhances the Therapeutic Activity of Mesenchymal Stromal Cells in Animal Models of Colitis. Stem Cells.

[77] Rafei M., Birman E., Forner K., Galipeau J. (2009). Allogeneic Mesenchymal Stem Cells for Treatment of Experimental Autoimmune Encephalomyelitis. Mol. Ther..

[78] Zheng G., Qiu G., Ge M., He J., Huang L., Chen P., Wang W., Xu Q., Hu Y., Shu Q. (2017). Human adipose-derived mesenchymal stem cells alleviate obliterative bronchiolitis in a murine model via IDO. Respir. Res..

[79] Han X., Yang Q., Lin L., Xu C., Zheng C., Chen X., Han Y., Li M., Cao W., Cao K. (2014). Interleukin-17 enhances immunosuppression by mesenchymal stem cells. Cell Death Differ..

[80] Polchert D., Sobinsky J., Douglas G.W., Kidd M., Moadsiri A., Reina E., Genrich K., Mehrotra S., Setty S., Smith B.J. (2008). IFN-γ activation of mesenchymal stem cells for treatment and prevention of graft versus host disease. Eur. J. Immunol..

[81] Liu G.-Y., Liu Y., Lu Y., Qin Y.-R., Di G.-H., Lei Y.-H., Liu H.-X., Li Y.-Q., Wu C., Hu X.-W. (2016). Short-term memory of danger signals or environmental stimuli in mesenchymal stem cells: Implications for therapeutic potential. Cell. Mol. Immunol..

[82] Sangiorgi B., Panepucci R.A. (2016). Modulation of Immunoregulatory Properties of Mesenchymal Stromal Cells by Toll-Like Receptors: Potential Applications on GVHD. Stem Cells Int..

[83] Qiu Y., Guo J., Mao R., Chao K., Chen B.-L., He Y., Zeng Z.-R., Zhang S.-H., Chen M.-H. (2016). TLR3 preconditioning enhances the therapeutic efficacy of umbilical cord mesenchymal stem cells in TNBS-induced colitis via the TLR3-Jagged-1-Notch-1 pathway. Mucosal Immunol..

[84] Waterman R.S., Tomchuck S.L., Henkle S.L., Betancourt A.M. (2010). A New Mesenchymal Stem Cell (MSC) Paradigm: Polarization into a Pro-Inflammatory MSC1 or an Immunosuppressive MSC2 Phenotype. PLoS ONE.

[85] Saeedi P., Halabian R., Fooladi A.A.I. (2019). Antimicrobial effects of mesenchymal stem cells primed by modified LPS on bacterial clearance in sepsis. J. Cell. Physiol..

[86] Ahn J.-S., Seo Y., Oh S.-J., Yang J.W., Shin Y.Y., Lee B.-C., Kang K.-S., Sung E.-S., Lee B.-J., Mohammadpour H. (2020). The activation of NLRP3 inflammasome potentiates the immunomodulatory abilities of mesenchymal stem cells in a murine colitis model. BMB Rep..

[87] Kurte M., Vega-Letter A.M., Luz-Crawford P., Djouad F., Noël D., Khoury M., Carrión F. (2020). Time-dependent LPS exposure commands MSC immunoplasticity through TLR4 activation leading to opposite therapeutic outcome in EAE. Stem Cell Res. Ther..

[88] Amati E., Sella S., Perbellini O., Alghisi A., Bernardi M., Chieregato K., Lievore C., Peserico D., Rigno M., Zilio A. (2017). Generation of mesenchymal stromal cells from cord blood: Evaluation of in vitro quality parameters prior to clinical use. Stem Cell Res. Ther..

[89] Neto M.D., Oliveira M.B., Mano J.F. (2019). Microparticles in Contact with Cells: From Carriers to Multifunctional Tissue Modulators. Trends Biotechnol..

[90] Zimmermann J.A., Hettiaratchi M.H., McDevitt T.C. (2016). Enhanced Immunosuppression of T Cells by Sustained Presentation of Bioactive Interferon-γ Within Three-Dimensional Mesenchymal Stem Cell Constructs. Stem Cells Transl. Med..

[91] Ranganath S.H., Tong Z., Levy O., Martyn K., Karp J.M., Inamdar M.S. (2016). Controlled Inhibition of the Mesenchymal Stromal Cell Pro-inflammatory Secretome via Microparticle Engineering. Stem Cell Rep..

[92] Ankrum J.A., Dastidar R.G., Ong J.F., Levy O., Karp J.M. (2014). Performance-enhanced mesenchymal stem cells via intracellular delivery of steroids. Sci. Rep..

[93] Varkouhi A.K., Monteiro A.P.T., Tsoporis J.N., Mei S.H.J., Stewart D.J., Dos Santos C.C. (2020). Genetically Modified Mesenchymal Stromal/Stem Cells: Application in Critical Illness. Stem Cell Rev. Rep..

[94] Kotterman M.A., Chalberg T.W., Schaffer D.V. (2015). Viral Vectors for Gene Therapy: Translational and Clinical Outlook. Annu. Rev. Biomed. Eng..

[95] Wu Z., Asokan A., Samulski R.J. (2006). Adeno-associated Virus Serotypes: Vector Toolkit for Human Gene Therapy. Mol. Ther..

[96] Yin H., Kanasty R.L., Eltoukhy A.A., Vegas A.J., Dorkin J.R., Anderson D.G. (2014). Non-viral vectors for gene-based therapy. Nat. Rev. Genet..

[97] Nakajima M., Nito C., Sowa K., Suda S., Nishiyama Y., Nakamura-Takahashi A., Nitahara-Kasahara Y., Imagawa K., Hirato T., Ueda M. (2017). Mesenchymal Stem Cells Overexpressing Interleukin-10 Promote Neuroprotection in Experimental Acute Ischemic Stroke. Mol. Ther. Methods Clin. Dev..

[98] Payne N.L., Sun G., McDonald C., Moussa L., Emerson-Webber A., Loisel-Meyer S., Medin J.A., Siatskas C., Bernard C.C. (2013). Human adipose-derived mesenchymal stem cells engineered to secrete IL-10 inhibit APC function and limit CNS autoimmunity. Brain Behav. Immun..

[99] Maiti P., Peruzzaro S., Kolli N., Andrews M., Gharaibeh A., Rossignol J., Dunbar G.L. (2019). Transplantation of mesenchymal stem cells overexpressing interleukin-10 induces autophagy response and promotes neuroprotection in a rat model of TBI. J. Cell. Mol. Med..

[100] Niu J., Yue W., Song Y., Zhang Y., Qi X., Wang Z., Liu B., Shen H., Hu X. (2014). Prevention of acute liver allograft rejection by IL-10-engineered mesenchymal stem cells. Clin. Exp. Immunol..

[101] Wang C., Lv D., Zhang X., Ni Z.-A., Sun X., Zhu C. (2018). Interleukin-10-Overexpressing Mesenchymal Stromal Cells Induce a Series of Regulatory Effects in the Inflammatory System and Promote the Survival of Endotoxin-Induced Acute Lung Injury in Mice Model. DNA Cell Biol..

[102] Manning E., Pham S., Li S., Vazquez-Padron R.I., Mathew J., Ruiz P., Salgar S.K. (2010). Interleukin-10 Delivery via Mesenchymal Stem Cells: A Novel Gene Therapy Approach to Prevent Lung Ischemia–Reperfusion Injury. Hum. Gene Ther..

[103] Payne N.L., Dantanarayana A., Sun G., Moussa L., Caine S., McDonald C., Herszfeld D., Bernard C.C., Siatskas C. (2012). Early intervention with gene-modified mesenchymal stem cells overexpressing interleukin-4 enhances anti-inflammatory responses and functional recovery in experimental autoimmune demyelination. Cell Adhes. Migr..

[104] Song S.Y., Hong J., Go S., Lim S., Sohn H.S., Kang M., Jung G., Yoon J., Kang M.L., Im G. (2020). Interleukin-4 Gene Transfection and Spheroid Formation Potentiate Therapeutic Efficacy of Mesenchymal Stem Cells for Osteoarthritis. Adv. Health Mater..

[105] Silva D.N., Souza B.S.F., Vasconcelos J.F., Azevedo C.M., Valim C.X.R., Paredes B.D., Rocha V.P.C., Carvalho G.B., Daltro P.S., Macambira S.G. (2018). Granulocyte-Colony Stimulating Factor-Overexpressing Mesenchymal Stem Cells Exhibit Enhanced Immunomodulatory Actions Through the Recruitment of Suppressor Cells in Experimental Chagas Disease Cardiomyopathy. Front. Immunol..

[106] Marin-Bañasco C., Benabdellah K., Melero-Jerez C., Oliver B., Pinto-Medel M.J., Hurtado-Guerrero I., De Castro F., Clemente D., Fernández O., Martin F. (2017). Gene therapy with mesenchymal stem cells expressing IFN-ß ameliorates neuroinflammation in experimental models of multiple sclerosis. Br. J. Pharmacol..

[107] Zheng Y.-B., Zhang X.-H., Huang Z.-L., Lin C.-S., Lai J., Gu Y.-R., Lin B.-L., Xie D.-Y., Xie S.-B., Peng L. (2012). Amniotic-Fluid–Derived Mesenchymal Stem Cells Overexpressing Interleukin-1 Receptor Antagonist Improve Fulminant Hepatic Failure. PLoS ONE.

[108] Xu J., Chen J., Li W., Lian W., Huang J., Lai B., Li L., Huang Z. (2020). Additive Therapeutic Effects of Mesenchymal Stem Cells and IL-37 for Systemic Lupus Erythematosus. J. Am. Soc. Nephrol..

[109] Martinez-Gonzalez I., Roca O., Masclans J.R., Moreno R., Salcedo M.-T., Baekelandt V., Cruz M.J., Rello J., Aran J.M. (2013). Human Mesenchymal Stem Cells Overexpressing the IL-33 Antagonist Soluble IL-1 Receptor–Like–1 Attenuate Endotoxin-Induced Acute Lung Injury. Am. J. Respir. Cell Mol. Biol..

[110] Meng X., Zheng M., Yu M., Bai W., Zuo L., Bu X., Liu Y., Xia L., Hu J., Liu L. (2019). Transplantation of CRISPRa system engineered IL10-overexpressing bone marrow-derived mesenchymal stem cells for the treatment of myocardial infarction in diabetic mice. J. Biol. Eng..

[111] Chen L., Lu F.-B., Chen D.-Z., Wu J.-L., Hu E.-D., Xu L.-M., Zheng M.-H., Li H., Huang Y., Jin X.-Y. (2018). BMSCs-derived miR-223-containing exosomes contribute to liver protection in experimental autoimmune hepatitis. Mol. Immunol..

[112] Wei Z., Qiao S., Zhao J., Liu Y., Li Q., Wei Z., Dai Q., Kang L., Xu B. (2019). miRNA-181a over-expression in mesenchymal stem cell-derived exosomes influenced inflammatory response after myocardial ischemia-reperfusion injury. Life Sci..

[113] Qu Y., Zhang Q., Cai X., Li F., Ma Z., Xu M., Lu L. (2017). Exosomes derived from miR-181-5p-modified adipose-derived mesenchymal stem cells prevent liver fibrosis via autophagy activation. J. Cell. Mol. Med..

[114] Jiang M., Wang H., Jin M., Yang X., Ji H., Jiang Y., Zhang H., Wu F., Wu G., Lai X. (2018). Exosomes from MiR-30d-5p-ADSCs Reverse Acute Ischemic Stroke-Induced, Autophagy-Mediated Brain Injury by Promoting M2 Microglial/Macrophage Polarization. Cell. Physiol. Biochem..

[115] Mei S.H.J., McCarter S.D., Deng Y., Parker C.H., Liles W.C., Stewart D.J. (2007). Prevention of LPS-Induced Acute Lung Injury in Mice by Mesenchymal Stem Cells Overexpressing Angiopoietin 1. PLoS Med..

[116] Sah S.K., Park K.H., Yun C.-O., Kang K.-S., Kim T.-Y. (2016). Effects of Human Mesenchymal Stem Cells Transduced with Superoxide Dismutase on Imiquimod-Induced Psoriasis-Like Skin Inflammation in Mice. Antioxid. Redox Signal..

[117] Yang J.W., Seo Y., Shin T.-H., Ahn J.-S., Oh S.-J., Shin Y.Y., Kang M.-J., Lee B.-C., Lee S., Kang K.-S. (2020). Extracellular Vesicles from SOD3-Transduced Stem Cells Exhibit Improved Immunomodulatory Abilities in the Murine Dermatitis Model. Antioxidants.

[118] Sims J.E., Smith D.E. (2010). The IL-1 family: Regulators of immunity. Nat. Rev. Immunol..

[119] De La Fuente-Núñez C., Lu T.K. (2017). CRISPR-Cas9 technology: Applications in genome engineering, development of sequence-specific antimicrobials, and future prospects. Integr. Biol..

[120] Hsu P.D., Lander E.S., Zhang F. (2014). Development and Applications of CRISPR-Cas9 for Genome Engineering. Cell.

[121] Manghwar H., Li B., Ding X., Hussain A., Lindsey K., Zhang X., Jin S. (2020). CRISPR/Cas Systems in Genome Editing: Methodologies and Tools for sgRNA Design, Off-Target Evaluation, and Strategies to Mitigate Off-Target Effects. Adv. Sci..

[122] Filho D.M., Ribeiro P.D.C., Oliveira L.F., Dos Santos A.L.R.T., Parreira R.C., Pinto M.C.X., Resende R.R. (2019). Enhancing the Therapeutic Potential of Mesenchymal Stem Cells with the CRISPR-Cas System. Stem Cell Rev. Rep..

[123] Shen Y., Zhang J., Yu T., Qi C. (2018). Generation of PTEN knockout bone marrow mesenchymal stem cell lines by CRISPR/Cas9-mediated genome editing. Cytotechnology.

[124] Zha S., Tay J.C.-K., Zhu S., Li Z., Du Z., Wang S. (2020). Generation of Mesenchymal Stromal Cells with Low Immunogenicity from Human PBMC-Derived β2 Microglobulin Knockout Induced Pluripotent Stem Cells. Cell Transplant..

[125] Hu X., Li L., Yu X., Zhang R., Yan S., Zeng Z., Shu Y., Zhao C., Wu X., Lei J. (2017). CRISPR/Cas9-mediated reversibly immortalized mouse bone marrow stromal stem cells (BMSCs) retain multipotent features of mesenchymal stem cells (MSCs). Oncotarget.

[126] Lee J., Bayarsaikhan D., Arivazhagan R., Park H., Lim B., Gwak P., Jeong G.-B., Lee J., Byun K., Lee B. (2019). CRISPR/Cas9 Edited sRAGE-MSCs Protect Neuronal Death in Parkinson’s Disease Model. Int. J. Stem Cells.

[127] Srifa W., Kosaric N., Amorin A., Jadi O., Park Y., Mantri S., Camarena J., Gurtner G.C., Porteus M. (2020). Cas9-AAV6-engineered human mesenchymal stromal cells improved cutaneous wound healing in diabetic mice. Nat. Commun..

[128] Furuhata Y., Nihongaki Y., Sato M., Yoshimoto K. (2017). Control of Adipogenic Differentiation in Mesenchymal Stem Cells via Endogenous Gene Activation Using CRISPR-Cas9. ACS Synth. Biol..

[129] Sun S., Xiao J., Huo J., Geng Z., Ma K., Sun X., Fu X. (2018). Targeting ectodysplasin promotor by CRISPR/dCas9-effector effectively induces the reprogramming of human bone marrow-derived mesenchymal stem cells into sweat gland-like cells. Stem Cell Res. Ther..

[130] Hoarau-Véchot J., Rafii A., Touboul C., Pasquier J. (2018). Halfway between 2D and Animal Models: Are 3D Cultures the Ideal Tool to Study Cancer-Microenvironment Interactions?. Int. J. Mol. Sci..

[131] Kapałczyńska M., Kolenda T., Przybyła W., Zajączkowska M., Teresiak A., Filas V., Ibbs M., Bliźniak R., Łuczewski Ł., Lamperska K. (2018). 2D and 3D cell cultures—A comparison of different types of cancer cell cultures. Arch. Med. Sci..

[132] Petrenko Y., Syková E., Kubinová Š. (2017). The therapeutic potential of three-dimensional multipotent mesenchymal stromal cell spheroids. Stem Cell Res. Ther..

[133] Ylöstalo J.H., Bartosh T.J., Coble K., Prockop D.J. (2012). Human Mesenchymal Stem/Stromal Cells Cultured as Spheroids are Self-activated to Produce Prostaglandin E2 that Directs Stimulated Macrophages into an Anti-inflammatory Phenotype. Stem Cells.

[134] Yeh H.-Y., Liu B.-H., Sieber M., Hsu S.-H. (2014). Substrate-dependent gene regulation of self-assembled human MSC spheroids on chitosan membranes. BMC Genom..

[135] Bartosh T.J., Ylostalo J.H., Bazhanov N., Kuhlman J., Prockop D.J. (2013). Dynamic compaction of human mesenchymal stem/precursor cells into spheres self-activates caspase-dependent IL1 signaling to enhance secretion of modulators of inflammation and immunity (PGE2, TSG6, and STC1). Stem Cells.

[136] Bartosh T.J., Ylöstalo J.H., Mohammadipoor A., Bazhanov N., Coble K., Claypool K., Lee R.H., Choi H., Prockop D.J. (2010). Aggregation of human mesenchymal stromal cells (MSCs) into 3D spheroids enhances their antiinflammatory properties. Proc. Natl. Acad. Sci. USA.

[137] Ylostalo J.H., Bartosh T.J., Tiblow A., Prockop D.J. (2014). Unique characteristics of human mesenchymal stromal/progenitor cells pre-activated in 3-dimensional cultures under different conditions. Cytotherapy.

[138] Regmi S., Pathak S., Thanh T.P., Nguyen T.T., Sung J.-H., Yook S., Kim J.O., Yong C.S., Choi I., Doh K.-O. (2019). Intraportally delivered stem cell spheroids localize in the liver and protect hepatocytes against GalN/LPS-induced fulminant hepatic toxicity. Stem Cell Res. Ther..

[139] Redondo-Castro E., Cunningham C.J., Miller J., Brown H., Allan S.M., Pinteaux E. (2018). Changes in the secretome of tri-dimensional spheroid-cultured human mesenchymal stem cells in vitro by interleukin-1 priming. Stem Cell Res. Ther..

[140] Zimmermann J.A., Mcdevitt T.C. (2014). Pre-conditioning mesenchymal stromal cell spheroids for immunomodulatory paracrine factor secretion. Cytotherapy.

[141] Chaicharoenaudomrung N., Kunhorm P., Noisa P. (2019). Three-dimensional cell culture systems as an in vitro platform for cancer and stem cell modeling. World J. Stem Cells.

[142] Regmi S., Seo Y., Young C.S., Kim H.-S., Jeong J.-H., Ahn J.-S., Pathak S., Acharya S., Nguyen T.T., Yook S. (2021). Heterospheroid formation improves therapeutic efficacy of mesenchymal stem cells in murine colitis through immunomodulation and epithelial regeneration. Biomaterials.

[143] Murphy K.C., Whitehead J., Falahee P.C., Zhou D., Simon S.I., Leach J.K. (2017). Multifactorial Experimental Design to Optimize the Anti-Inflammatory and Proangiogenic Potential of Mesenchymal Stem Cell Spheroids. Stem Cells.

[144] Chen Y., Shu Z., Qian K., Wang J., Zhu H. (2019). Harnessing the Properties of Biomaterial to Enhance the Immunomodulation of Mesenchymal Stem Cells. Tissue Eng. Part B Rev..

[145] Follin B., Juhl M., Cohen S., Pedersen A.E., Gad M., Kastrup J., Ekblond A. (2015). Human adipose-derived stromal cells in a clinically applicable injectable alginate hydrogel: Phenotypic and immunomodulatory evaluation. Cytotherapy.

[146] Barminko J., Kim J.H., Otsuka S., Gray A., Schloss R., Grumet M., Yarmush M.L. (2011). Encapsulated mesenchymal stromal cells for in vivo transplantation. Biotechnol. Bioeng..

[147] Zanotti L., Sarukhan A., Mancuso F., Arato I., Golemac M., Jonjic N., Biondi A., Calafiore R., Locati M., D’Amico G. (2012). Encapsulated mesenchymal stem cells for in vivo immunomodulation. Leukemia.

[148] Stucky E.C., Schloss R.S., Yarmush M.L., Shreiber D.I. (2015). Alginate micro-encapsulation of mesenchymal stromal cells enhances modulation of the neuro-inflammatory response. Cytotherapy.

[149] Hanson S.E., King S.N., Kim J., Chen X., Thibeault S.L., Hematti P. (2011). The Effect of Mesenchymal Stromal Cell–Hyaluronic Acid Hydrogel Constructs on Immunophenotype of Macrophages. Tissue Eng. Part A.

[150] Li J., Chen T., Huang X., Zhao Y., Wang B., Yin Y., Cui Y., Zhao Y., Zhang R., Wang X. (2018). Substrate-independent immunomodulatory characteristics of mesenchymal stem cells in three-dimensional culture. PLoS ONE.

[151] Darnell M., Gu L., Mooney D. (2018). RNA-seq reveals diverse effects of substrate stiffness on mesenchymal stem cells. Biomaterials.

[152] Gómez-Aristizábal A., Kim K.-P., Viswanathan S. (2016). A Systematic Study of the Effect of Different Molecular Weights of Hyaluronic Acid on Mesenchymal Stromal Cell-Mediated Immunomodulation. PLoS ONE.

[153] Su N., Gao P.-L., Wang K., Wang J.-Y., Zhong Y., Luo Y. (2017). Fibrous scaffolds potentiate the paracrine function of mesenchymal stem cells: A new dimension in cell-material interaction. Biomaterials.

[154] Wan S., Fu X., Ji Y., Li M., Shi X., Wang Y. (2018). FAK- and YAP/TAZ dependent mechanotransduction pathways are required for enhanced immunomodulatory properties of adipose-derived mesenchymal stem cells induced by aligned fibrous scaffolds. Biomaterials.

[155] Li L.-M., Han M., Jiang X.-C., Yin X.-Z., Chen F., Zhang T.-Y., Ren H., Zhang J.-W., Hou T.-J., Chen Z. (2017). Peptide-Tethered Hydrogel Scaffold Promotes Recovery from Spinal Cord Transection via Synergism with Mesenchymal Stem Cells. ACS Appl. Mater. Interfaces.

[156] García J.R., Quirós M., Han W.M., O’Leary M.N., Cox G.N., Nusrat A., García A.J. (2019). IFN-γ-tethered hydrogels enhance mesenchymal stem cell-based immunomodulation and promote tissue repair. Biomaterials.

[157] Mao A.S., Özkale B., Shah N.J., Vining K.H., Descombes T., Zhang L., Tringides C.M., Wong S.-W., Shin J.-W., Scadden D.T. (2019). Programmable microencapsulation for enhanced mesenchymal stem cell persistence and immunomodulation. Proc. Natl. Acad. Sci. USA.

[158] Tsou Y.-H., Khoneisser J., Huang P.-C., Xueqing X. (2016). Hydrogel as a bioactive material to regulate stem cell fate. Bioact. Mater..

[159] Fischer U.M., Harting M.T., Jimenez F., Monzon-Posadas W.O., Xue H., Savitz S.I., Laine G.A., Cox C.S. (2009). Pulmonary Passage is a Major Obstacle for Intravenous Stem Cell Delivery: The Pulmonary First-Pass Effect. Stem Cells Dev..

